# Amphipod species in European and nearby seas

**DOI:** 10.3897/BDJ.14.e186195

**Published:** 2026-04-01

**Authors:** Antonina Badalucco, Sabrina Lo Brutto, Jean-Claude Dauvin, Mark John Costello

**Affiliations:** 1 Department of Earth and Marine Sciences (DiSTeM), University of Palermo, Palermo, Italy Department of Earth and Marine Sciences (DiSTeM), University of Palermo Palermo Italy; 2 National Biodiversity Future Center (NBFC), Palermo, Italy National Biodiversity Future Center (NBFC) Palermo Italy; 3 CNRS UMR 6143 M2C, Caen, France CNRS UMR 6143 M2C Caen France; 4 University of Caen Normandy, Caen, France University of Caen Normandy Caen France; 5 Faculty of Biosciences and aquaculture, Nord University, Bodø, Norway Faculty of Biosciences and aquaculture, Nord University Bodø Norway

**Keywords:** Amphipoda, Peracarida, European Seas, North-African Atlantic, species occurrence

## Abstract

**Background:**

Data on the geographical distribution of species underpin nature conservation planning, natural resource management, environmental assessments and fundamental understanding of the natural world. Here, we provide a dataset on Amphipoda species reported in the scientific literature to be present in European and adjacent seas.

**New information:**

In this dataset, each record is associated with taxonomic information (i.e. species, genus, family, order, class, phylum, kingdom) and sampling event details including date, location and geographical coordinates (longitude and latitude). The occurrence records included in the dataset were collected from 1936 to 2024. The study area encompassed the north-eastern Atlantic Ocean (from the region of northern Norway to the Cape Verde Archipelago, including the Icelandic waters), the Baltic Sea, the Mediterranean Sea and the Black Sea. These regions were characterised by fragmented species distribution information, dispersed across multiple national maritime territories and pronounced variation in key environmental parameters, including salinity, temperature and bathymetry. An inventory of 18,559 records, corresponding to 1,238 species, is presented here.

## Introduction

Amphipods are small crustaceans comprising more than 10,000 valid species distributed across 242 families and 1,766 genera, inhabiting marine, freshwater and semi-terrestrial environments (Horton et al. 2023). Recent taxonomic revisions recognise six suborders: Amphilochidea, Colomastigidea, Hyperiidea, Hyperiopsidea, Pseudingolfiellidea and Senticaudata (Lowry and Myers 2017). It is estimated that one-third of extant amphipod species remain to be formally described (Arfianti et al. 2018). Amphipods exhibit laterally compressed, segmented bodies consisting of a cephalon, pereon and pleon (Conlan 1991, Arfianti and Costello 2020a). Their specialised appendages support a wide range of ecological niches, including free-living, burrowing, commensal and parasitic lifestyles (Scipione 2013). Sexual dimorphism is pronounced, with males commonly bearing enlarged antennae and gnathopods (e.g. Lo Brutto et al. (2022)). Reproduction is direct and dioecious, with females brooding embryos in a ventral marsupium; life-history traits are strongly influenced by environmental parameters, such as temperature and food availability (Wildish 2012, Costa et al. 2021). Ecologically, amphipods occupy several trophic levels, functioning as grazers, detritivores, scavengers, filter feeders and prey for vertebrates (Scipione 2013, Dauvin 2024, Ritter and Bourne 2024). Their sensitivity to contaminants — including oil spills, wastewater effluents, pharmaceuticals and microplastics — makes them effective bioindicators in ecotoxicological assessment (Dauvin 1987, Borja et al. 2000, de-la-Ossa-Carretero et al. 2012, Lo Brutto et al. 2021, Ritter and Bourne 2024). They also act as ecosystem engineers, influencing sediment stability and geochemistry through bioturbation (Brandt et al. 2023). Functional-group analyses have proven useful in revealing ecological patterns: for instance, herbivorous and detritivorous species dominate shallow *Posidonia oceanica* meadows, whereas omnivores and carnivores increase at greater depths (Scipione 2013).

At a regional scale, amphipods display remarkable diversity and endemicity across both marine and inland waters. For instance, in Italian seas, 500 species have been recorded, accounting for 76.7% of all Mediterranean species (Badalucco et al. 2024). In northern Adriatic regions, national inventories, such as Slovenia’s checklist, highlighted the ecological and species richness of the group, documenting 198 species across 41 families, including 26 endemics mostly restricted to subterranean freshwater systems (Fišer et al. 2021). Amphipod diversity is also pronounced in extreme or structurally complex habitats, such as marine and anchialine caves (Navarro-Barranco et al. 2023). Symbiotic associations are frequent, exemplified by the commensal relationship between *Leucothoe richiardii* and the ascidian *Phallusia mammillata* (Arduini et al. 2023) or the species which spend their life in the mantle cavity of subtidal bivalves (Vader and Tandberg 2013).

Globally, taxonomic efforts have been consolidated through the World Amphipoda Database (WAD), part of the World Register of Marine Species (WoRMS) (Costello et al. 2013, Horton et al. 2023). The WAD, in its current format, originated in 2010 from the integration of several previously developed taxonomic resources (Horton et al. 2023). It resulted from the merger of the World Amphipoda List, compiled over many years by Jim Lowry, with the amphipod component of the European Register of Marine Species (ERMS) (Bellan-Santini and Costello 2001, Costello and Emblow 2001). The WAD standardises nomenclature, synonymies and bibliographic authorities, with the objective of integrating species traits and distributional data (Horton et al. 2023).

Overall, amphipods represent a key taxon for ecological and biogeographical studies (Costello and Myers 1991, Arfianti and Costello 2020b). Their morpho-functional diversity enables colonisation of virtually all aquatic habitats, from the groundwater systems to the deep sea and their assemblages are primarily structured by depth, substratum and temperature (Iaciofano et al. 2024). As such, ongoing range shifts associated with ocean warming and biological invasions underscore their value as indicators of global environmental change.

In this article, we describe a dataset containing georeferenced records of amphipod occurrences across European, North Atlantic and African marine and transitional waters, providing a comprehensive resource to support future biodiversity assessments and biogeographical analyses. We advanced the European Register of Marine Species (ERMS; Bellan-Santini and Costello (2001)) providing georeferenced distribution of species listed.

## General description

### Purpose

The dataset collects all the nominal Amphipoda species retrieved from the scientific literature and the respective distribution across marine European and nearby areas, including the north Atlantic African and the Mediterranean African waters. In this dataset, each record is associated with taxonomic information (i.e. species, genus, family, order, class, phylum, kingdom) and sampling event details including date, location and geographical coordinates. When literature sources did not provide geographical coordinates, the occurrences were placed either as the closest point to the locality or the georeferenced point in the middle of the area. All the coordinates were to ensure that none of them was laid on land. The occurrence records included in the dataset were collected from 1936 to 2024. The nomenclature of all species has been updated in accordance with the World Amphipoda Database (WAD; Horton et al. (2026)). Species occurring in benthic and pelagic, brackish, near-freshwater or transitional zones have also been included to ensure a complete representation of species occupying the interface between marine and freshwater environments. The dataset includes over 18,559 records distributed across 1,018 coordinates. This collection of data provides information from the Mediterranean area and north-eastern Atlantic waters, which could be used by the scientific community in further research studies and by stakeholders and policy-makers to define conservation priorities and improve monitoring programmes.

## Project description

### Title

Amphipod species in European marine waters

### Personnel

Antonina Badalucco, Sabrina Lo Brutto, Jean-Claude Dauvin, Mark John Costello

### Study area description

The study area associated with this project include a wide geographic coverage, from the north-eastern Atlantic Ocean to the Atlantic African waters and to the Mediterranean and Black Sea.

### Design description

The dataset collects all the nominal Amphipoda species retrieved from the scientific literature and the respective distribution across marine European and nearby waters. In this dataset, each record is associated with taxonomic information and sampling event details including date, location and geographical coordinates. The occurrence records included in the dataset were collected from 1936 to 2024.

### Funding

Project funded under: (a) the National Recovery and Resilience Plan (PNRR), Mission 4 Component 2 Investment 1.4, NextGenerationEU. Award Number: Project code CN_00000033, Concession Decree No. 1034 of 17 June 2022 adopted by the Italian Ministry of University and Research, CUP B73C22000790001, Project title “National Biodiversity Future Center – NBFC”; (b) the MPA Europe project, funded under the Horizon Europe Grant Agreement no. 101059988.

## Sampling methods

### Step description

Data were gathered from published scientific literature. The bibliographic search was conducted in the period 2024-2025, using Google Scholar's “Advanced Search” tool, using the keywords “amphipod*”, “peracarid*”, “crustacea*”, “checklist" in combination with the names of European countries and seas, extending the search to the North African coast. About 150 articles have been selected and consulted, avoiding redundant information. The references are listed in the database (attribute #associatedReferences). Species occurring in benthic and pelagic, brackish, near-freshwater and transitional ecosystems have been included to ensure a complete representation of species occupying the interface between marine and freshwater environments.

Data were organised following the Darwin Core Standard. The taxonomic information has been updated in accordance with the World Amphipoda Database provided by World Marine Species Database (https://www.marinespecies.org/amphipoda/).

## Geographic coverage

### Description

The study area encompasses the north-eastern Atlantic Ocean (from the region of northern Norway to the Cape Verde Archipelago, including the Icelandic waters), the Mediterranean Sea and the Black Sea.

**Figure 1.**
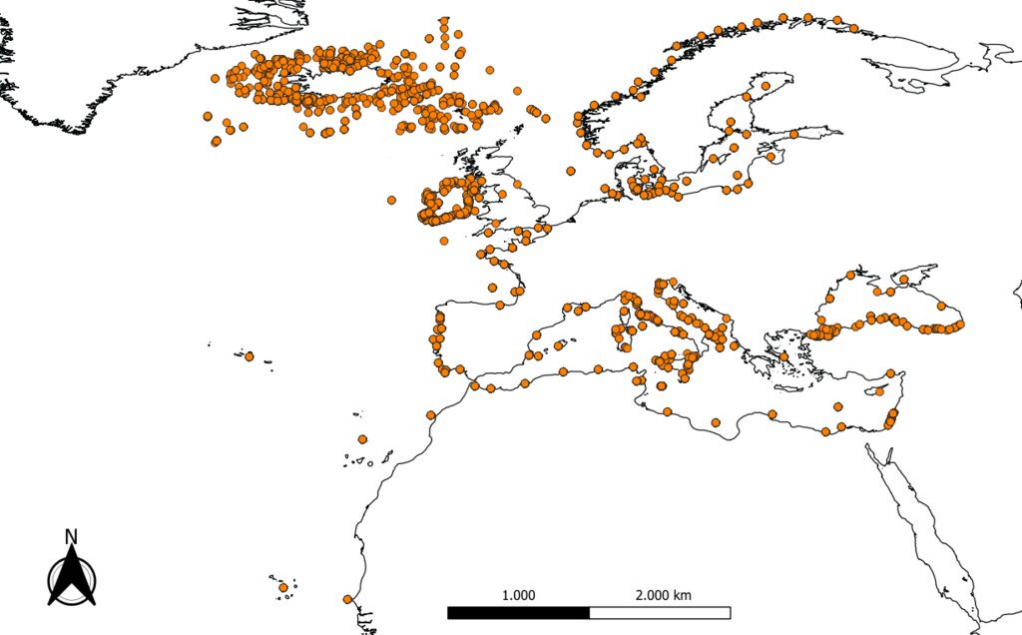
Map of the study area showing the total of 1,018 geographic coordinates of the sampling sites, generated using QGIS software (QGIS 2025).

Species were registered at least at the country level, according to political boundaries and the dataset includes records from 33 countries. The covered marine area extends across the European Atlantic Ocean and the North-Atlantic waters from the Strait of Gibraltar to Senegal, including the Cape Verde, Canaria, Madeira and Azores archipelagos, the Mediterranean Sea and the Black Sea, encompassing a wide range of marine and transitional environments (Fig. 1). Although the dataset focuses primarily on European waters, it also includes adjacent Atlantic and Mediterranean areas that are not strictly within the political boundaries of Europe, but are ecologically connected to the European marine region. The dataset comprises information collected from more than 300 localities, corresponding to 1,018 geographic coordinates. The coordinates defined the maximum geographical extent of the dataset, covering coastal areas throughout the north-eastern Atlantic, the Mediterranean and the Black Sea regions. In western Europe, numerous records were concentrated along the Atlantic coasts of France, Spain and Portugal, including the Bay of Biscay and the Gulf of Cádiz (16% of all records). Records were also reported from the Canary Islands and the Azores Archipelago (2%). Along the Irish and British coasts, a high density of records occurred in the North Sea, Irish Sea and English Channel, extending northwards to Shetland (2%). Similarly, Scotland and Ireland showed high sampling effort (9%). Towards the North Atlantic, an extensive occurrence data coverage was observed along the Norwegian and Icelandic coasts and in the Faroes (20%). In the Baltic Sea, occurrence data were more scattered, distributed along the coasts of Denmark, Sweden and Finland, representing transitional and brackish-water environments, characterised by low salinity habitats (5%). In southern Europe, a high richness of records was concentrated within the Mediterranean Sea (34%). The western and central Mediterranean represented a significant part of the Mediterranean records (22%), with numerous records from the Spanish, French, Tunisian, Algerian and Morocco coasts, including the Balearic Islands and Corsica. The main occurrence records were distributed along the Italian coastline, covering the Ligurian, Tyrrhenian, Ionian and Adriatic Seas. Sampling extended from the northernmost localities of the Italian Friuli Venezia Giulia region to the southernmost sites in Sicily and longitudinally from Sardinia in the west to the Apulia Region in the east, representing one of the most intensively sampled regions of the Mediterranean (18%). These areas host a mix of Atlanto-Mediterranean endemic and non-native taxa. In the eastern Mediterranean, data points were concentrated along the Greek archipelagos, the Aegean and Ionian Seas and along the Turkish and Cypriot coasts (2%). Further records were located in the Levantine Basin (4%), particularly along Israel and Lebanon, documenting amphipod assemblages typical of oligotrophic, warm-temperate waters. Some regions remained under-represented, including portions of the eastern Adriatic, eastern part of the North African and Black Sea coasts (Fig. 1). As in most biodiversity datasets, this uneven spatial distribution reflects differences in sampling effort, research intensity and data accessibility.

Overall, the compiled dataset not only highlights regions of high sampling efforts, but also identifies geographic gaps. Such information can support environmental managers and policy-makers in prioritising future biomonitoring and conservation efforts, particularly in data-poor or ecologically sensitive habitats and areas.

### Coordinates

14.833 and 70.833 Latitude; -31.37666 and 41.514 Longitude.

## Taxonomic coverage

### Description

The present dataset includes the valid scientific names of all the detected marine amphipod species. The taxonomic nomenclature follows the World Register of Marine Species (WoRMS) to ensure consistency and accuracy across taxa. In addition to the valid names, the dataset also includes two supplementary columns: one listing the specific names as they appeared in the bibliographic sources and another reporting the authority, in accordance with WAD database.

Overall, the dataset contains 18,559 records, corresponding to 1,238 species, 128 families and 422 genera. Amongst the six suborders currently recognised within the order Amphipoda, five are represented in the European marine waters: Amphilochidea, Colomastigidea, Hyperiidea, Senticaudata and Hyperiopsidea. Two records belonging to an *incertae sedis* species, *Paramphithoe hystrix*, are included, whose subordinal placement remains uncertain. The suborder Senticaudata accounted for the highest number of records, followed by Amphilochidea. However, Amphilochidea showed the highest species richness, indicating a broad taxonomic diversity within this group. At the family level, the five families with the largest number of records were Ampeliscidae (5.86%), Oedicerotidae (5.61%), Caprellidae (5.51%), Corophiidae (5.02%) and Gammaridae (4.83%). The five most represented genera in terms of records were *Ampelisca* (4.98%), *Gammarus* (3.07%), *Caprella* (2.99%), *Harpinia* (2.48%) and *Microdeutopus* (2.09%).

## Temporal coverage

### Notes

1936-01-01 through 2024-12-31

## Usage licence

### Usage licence

Other

### IP rights notes

This work is licensed under a Creative Commons Attribution (CC-BY) 4.0 License.

## Data resources

### Data package title

Amphipod species in European marine waters

### Resource link

https://doi.org/10.15468/bac3bs

### Alternative identifiers

www.gbif.org/dataset/1260320d-5eb6-475b-bf4a-a60cf3f9e3e1; https://cloud.gbif.org/eca/resource?r=european_marine_amphipods; https://metadatacatalogue.lifewatch.eu/srv/eng/catalog.search#/metadata/ac2350c3-b912-4ce6-89bb-c922e15ec2cc

### Number of data sets

1

### Data set 1.

#### Data set name

Amphipod species in European marine waters

#### Data format

Darwin Core

#### Description

The dataset comprises all the nominal Amphipoda species retrieved from the scientific literature and the respective distribution across European and adjacent seas. In this dataset, each record is associated with taxonomic information (i.e. species, genus, family, order, class, phylum, kingdom) and sampling event details including date, location and geographical coordinates (Badalucco et al. 2026). When publications did not provide geographical coordinates, the occurrences were placed at the point closest to the sampling site (Table 1). The occurrence records included in the dataset were collected from 1936 to 2024.

## Figures and Tables

**Table 1.** Description of the data table attributes.

**Attribute name**	**Attribute definition**
institutionCode	The name (or acronym) in use by the institution having custody of the object(s) or information referred to in the record.
basisOfRecord	The specific nature of the data record.
occurrenceID	An identifier for the dwc:Occurrence (as opposed to a particular digital record of the dwc:Occurrence). In the absence of a persistent global unique identifier, construct one from a combination of identifiers in the record that will most closely make the dwc:occurrenceID globally unique.
associatedReferences	A list (concatenated and separated) of identifiers (publication, bibliographic reference, global unique identifier, URI) of literature associated with the dwc:Occurrence.
projectTitle	A list (concatenated and separated) of titles or names for projects that contributed to a dwc:Event.
eventDate	The date-time or interval during which a dwc:Event occurred. For occurrences, this is the date-time when the dwc:Event was recorded. Not suitable for a time in a geological context.
higherGeography	A list (concatenated and separated) of geographic names less specific than the information captured in the dwc:locality term.
waterBody	The name of the water body in which the dcterms:Location occurs.
locality	The specific description of the place.
decimalLatitude	The geographic latitude (in decimal degrees, using the spatial reference system given in dwc:geodeticDatum) of the geographic centre of a dcterms:Location. Positive values are north of the Equator, negative values are south of it. Legal values lie between -90 and 90, inclusive.
decimalLongitude	The geographic longitude (in decimal degrees, using the spatial reference system given in dwc:geodeticDatum) of the geographic centre of a dcterms:Location. Positive values are east of the Greenwich Meridian, negative values are west of it. Legal values lie between -180 and 180, inclusive.
geodeticDatum	The ellipsoid, geodetic datum or spatial reference system (SRS) upon which the geographic coordinates given in dwc:decimalLatitude and dwc:decimalLongitude are based.
verbatimIdentification	A string representing the taxonomic identification as it appeared in the original record.
acceptedNameUsageID	An identifier for the name usage (documented meaning of the name according to a source) of the currently valid (zoological) or accepted (botanical) taxon.
scientificName	The full scientific name, with authorship and date information if known. When forming part of a dwc:Identification, this should be the name in lowest level taxonomic rank that can be determined. This term should not contain identification qualifications, which should instead be supplied in the dwc:identificationQualifier term.
acceptedNameUsage	The full name, with authorship and date information if known, of the currently valid (zoological) or accepted (botanical) dwc:Taxon.
kingdom	The full scientific name of the kingdom in which the dwc:Taxon is classified.
phylum	The full scientific name of the phylum or division in which the dwc:Taxon is classified.
class	The full scientific name of the class in which the dwc:Taxon is classified.
order	The full scientific name of the order in which the dwc:Taxon is classified.
family	The full scientific name of the family in which the dwc:Taxon is classified.
genus	The full scientific name of the genus in which the dwc:Taxon is classified.
taxonRank	The taxonomic rank of the most specific name in the dwc:scientificName.
scientificNameAuthorship	The authorship information for the dwc:scientificName formatted according to the conventions of the applicable dwc:nomenclaturalCode.
